# The science is in the data

**DOI:** 10.1107/S2052252517013690

**Published:** 2017-10-06

**Authors:** John R. Helliwell, Brian McMahon, J. Mitchell Guss, Loes M. J. Kroon-Batenburg

**Affiliations:** a School of Chemistry, University of Manchester, Manchester M13 9PL, England; b International Union of Crystallography, 5 Abbey Square, Chester CH1 2HU, England; c School of Life and Environmental Sciences, The University of Sydney, Sydney, NSW 2006, Australia; d Crystal and Structural Chemistry, Bijvoet Center for Biomolecular Research, Utrecht University, Padualaan 8, CH 3584 Utrecht, The Netherlands

**Keywords:** raw diffraction data, sharing raw data and its reuse, open science, education, crystallographic science case studies

## Abstract

Understanding published research results should be through one’s own eyes and include the opportunity to work with raw diffraction data to check the various decisions made in the analyses by the original authors. Today, preserving raw diffraction data is technically and organizationally viable at a growing number of data archives, both centralized and distributed, which are empowered to register data sets and obtain a preservation descriptor, typically a ‘digital object identifier’. This introduces an important role of preserving raw data, namely understanding where we fail in or could improve our analyses. Individual science area case studies in crystallography are provided.

## Abstract 2

Understanding published research results should be through one’s own eyes and include the raw diffraction data, an option that has recently become viable at various data archives. Preserving and sharing raw diffraction data will allow challenging data cases in crystallography to be more expeditiously tackled.

## 1. Introduction   

### 1.1. The significance of all sorts of scientific data   

The meaning of the title of this article seems almost self-evident. For scientific inquiry, the ‘data’ are what we collect to explore nature, to test hypotheses and to suggest novel properties and mechanisms, as well as make ‘findings of fact’. Yet ‘data’ is a very broad term. In crystallographic structure experiments, it may refer to the ‘raw’ data, such as diffraction images collected at the diffractometer (although even these are not truly ‘raw’, inasmuch as they are captured according to the electronics and mechanical properties of the detector, with whatever limitations or shortcomings are inherent to that particular device). It may also refer to the ‘processed’ data – for example merged structure factors – that result from calibration, reduction and other manipulation of the original images, and that constitute the material for structure solution and model refinement. The term ‘data’ is also used for the itemized description of the derived structural model itself (as in the coordinate sets and anisotropic displacement parameters stored in structural databases).

In all of these categories, crystallographic science resides. With raw diffraction data sets, we capture as much information as we can about the atoms of a crystal *in situ*. With the processed diffraction data sets, we retain an averaged description of the structural units in the crystal, but we may have ignored diffusely scattered intensities that contain information about disorder or large-scale correlations or we may have ignored a second crystal lattice, as in the case of pseudo-merohedral twinning. By the time we consider derived structure models, we have largely idealized a molecular structure or a ‘typical’ atomic environment. At each step, our level of abstraction is (usually) appropriate to the study at hand. However, the success of crystallographic hardware and software occasionally lulls us into a false sense of security, a slight tendency to forget the full complexities of nature that could be teased out from a closer inspection of the diffraction data set that we are examining.

Crystallography has a strong tradition of data sharing, and it is not necessary to labour the point that close and critical reanalysis of experimental data has frequently led to improvements in derived structural models (see, for example, Marsh *et al.*, 2002 ▸). In the field of chemical crystallography, there are exemplary behaviours such as that of *Acta Crystallographica Section C*, led by its Editor from 1993 to 1999, Sydney Hall (the winner of the CODATA 2014 International Data Prize), where referees and editor are provided with underpinning data and the submitted article. Thus, an accepted article has the structure factors and coordinates attached to it as ‘versions of record’ of the data. Subsequently, a chemical crystal structure database [*e.g.* Cambridge Structural Database (CSD), Crystallography Open Database (COD), Inorganic Crystal Structure Database (ICSD), International Centre for Diffraction Data (ICDD)] can derive great benefit from this due care and attention of referees and editor and harvest these versions of record of the data and article.

### 1.2. The particular importance of raw data   

Here, we focus on the value of re-examining and reusing raw data sets. Access to the raw diffraction data would allow the user/reader of crystallographic results to see directly every calculation choice made by the original researchers, and to take a different calculation route if they wish.

In a recent Topical Review, three of us have demonstrated how preserving raw diffraction data sets is now technically and organizationally viable at a growing number of digital data archives (Kroon-Batenburg *et al.*, 2017 ▸). We have argued that such archives, both centralized and distributed, should be empowered to register data sets and obtain a preservation descriptor. We note the large and growing proportion of cases in which this is the international standard ‘digital object identifier’ (DOI) maintained by a distributed network of accredited registration agencies (International Organization for Standardization, 2012 ▸). The longer-term availability of raw diffraction data is becoming more common, and we believe it is essential that an orderly infrastructure based on standards such as the DOI evolves to harmonize the emerging archives. The seminal articles discussing raw data preservation in the context of the Store.Synchrotron (Meyer *et al.*, 2014 ▸), Integrated Resource for Reproducibility in Macromolecular Crystallography (Grabowski *et al.*, 2016 ▸) and Structural Biology Data Grid (Meyer *et al.*, 2016 ▸) initiatives provide substantial support for this approach.

A number of drivers for data archiving in the wider scientific world have been identified by Kroon-Batenburg *et al.* (2017 ▸), including funding-body mandates for formal research data-management policies. Among these drivers we single out the future research vision based on ‘*Open Innovation, Open Science and Open to the World’* described in the European Union’s book (Moedas, 2016 ▸). This arises from the desire of science policy makers such as the European Union and the USA National Institutes of Health to speed up science discovery for urgent societal problems such as the improved treatment of disease and the mitigation of environmental pollution. Facilitating early data sharing before publication is a key part of this new ‘Open Science’ vision. The European Open Science Cloud programme (https://ec.europa.eu/research/openscience/index.cfm?pg=open-science-cloud) is providing tools and guidelines for Open Science in order to promote the crowdsourcing of solutions to urgent societal problems. Among its components may be counted the CERN-hosted Zenodo archive, which provides an open repository for scientific data sets in any field. Currently, it will accept ‘small’ data sets (<50 GB) free of charge from anywhere in the world, and it is currently being used by a relatively small number of crystallographers to make their raw diffraction data sets available.

We cannot know whether Zenodo will remain unique, or whether the growing pressure by funding bodies on researchers to archive their supporting experimental data will be met by commercial providers. Overall, however, the feasibility of the deposition of and then open access to raw experimental data sets underpinning publications is now greatly facilitated by the data archives such as those mentioned above, as well as those at the central experimental facilities (synchrotron radiation, X-ray lasers and neutron sources).

There remain practical challenges associated with data volumes and network bandwidth. The newest detectors generate quantities of data that take crystallography firmly into the ‘Big Data’ era (although we are still far from matching the data volumes of the radio astronomers with their Square Kilometre Array project). In a previous publication (Tanley, Schreurs *et al.*, 2013 ▸) we described the network-transfer times for moving approximately 10 GB-scale raw diffraction data sets between Manchester and Utrecht Universities; initially this took 5 d (although it could have been optimized to a day or so). Recent experience in retrieving data from the Structural Biology Grid (Meyer *et al.*, 2016 ▸) demonstrated that 15 GB of data can now be transferred in 30 min. The capacity of data-transfer networks is thus keeping pace to some extent with the growth in volume. Nevertheless, wherever possible, it is a good idea to archive the raw diffraction data near to where the data are measured, and thereby harness even faster local networks.

Another important role for preserving raw diffraction data applies to cases where we have diffraction data but no publication. These can help us to understand better where we fail in our analyses and no discovery results. For these challenging cases, the sharing of the raw diffraction data should be made open, not least where taxpayer funds have been applied.

Examples of these unfortunately cannot contribute to the case studies here, as they are not known about, except to the individual principal investigators (PIs) who hold these close to their laboratories or collaborators. Instead, in this article we describe individual case studies that we have found from the literature and that are drawn from widely different areas of crystallographic research and applications, in effect spanning the range of the IUCr’s various scientific Commissions. These publications either show directly the benefits of raw data preservation and reuse, or are direct examples in which a complete repeat of sample production through to new analyses and results occurred, which is clearly inefficient. Thus, preserving raw diffraction data would be a more secure way of protecting our crystallographic research enterprise.

In organizing our discussion by technique, we aim to demonstrate that the issues are relevant across the entire range of activities that fall under the existing IUCr Commissions. A fair proportion of the examples are from macromolecular crystallography, but we hope that this article will prompt the reporting of more need-for-raw-data cases from other fields. We note also that our case studies fall into three broad categories: those where data sharing is beneficial, those where data preservation is important in allowing further progress and those where the absence of data is a significant problem (Table 1 ▸).

## 2. Some definitions and the scope of this article   

In single-crystal structure analysis the ‘raw’ data is understood to mean the diffraction images, although to some extent these are already processed, *e.g.* distortion or flood-field corrected. The raw data can also be referred to as the ‘primary’ data, another perfectly acceptable term. The processing of these diffraction images leads to prediction of where the Bragg reflections intercept the detector (the Bragg spots) and their intensities are estimated; this step includes, firstly, determination of the unit-cell parameters of the crystal. From these processed diffraction data a molecular model is determined and refined. The finalized coordinates and atomic displacement parameters of the atoms in the model are termed the derived data. In crystal structure analysis, the probes and methods span the use of X-rays, neutrons and electrons, and the above descriptors (raw, processed and derived data) apply to each.

Other types of experimental sample, besides a single crystal, occur, *i.e.* a powder, a fibre, a surface, an amorphous solid or a liquid or gas. No real-world crystal or other material sample conforms in practice to an idealized model, and interpretation of a diffraction pattern or other structural experimental data set must take care not to discard significant features that describe the actual sample but are at variance with an idealized model. In the terms of this article, and at some risk of an overly generalized definition, the experimental ‘raw’ diffraction data for each of these non-single-crystal diffraction experiments constitute ‘the data’ as there are no intermediate processed data. The whole diffraction image leads directly to a derived molecular model and its structural dynamics. Such raw data are sometimes termed the ‘primary’ data, as mentioned above. In the case of a powder diffraction pattern the full two-dimensional diffraction pattern is, in the ideal sample case, reducible to a one-dimensional diffraction profile.

‘Real samples’ studied by diffraction need not be in any of the idealized states of matter mentioned above. Thus, a single crystal can have a variety of imperfections or dynamical states, either short-range within a unit cell or spanning many unit cells, which leads to ‘diffuse scattering’ underneath the Bragg peaks or between the Bragg peaks. Obviously, therefore, the raw diffraction images are needed for the study of the underlying disorder and dynamics. Recent extensive reviews of the methods of diffuse scattering and of the ‘pair distribution function (PDF)’ are given by Welberry & Weber (2016 ▸) and Billinge (2018 ▸), respectively.

The scope of this article is restricted to single-crystal and powder structure analyses from diffraction data.

## 3. A little history about raw diffraction data preservation and access   

It has been envisaged for a long time (Strickland *et al.*, 2008 ▸) that the preservation of and access to raw diffraction data is important, but technically and organizationally challenging; quoting from Strickland *et al.* (2008 ▸):

Ideally, the full scientific record should provide access to the raw data … the IUCr is beginning to consider longer-term approaches to archiving the raw data.

The publication of raw diffraction data has one of its earliest exemplars in Lawrence Bragg’s publication on the crystal structures of the alkali halides, with an extensive number of his own ‘Laue diffraction photographs’, measured in Cambridge, included in his article (Bragg, 1913 ▸).

**Figure 1:**
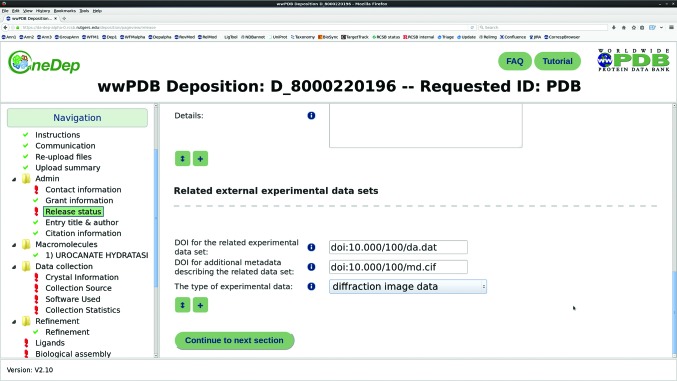
The wwPDB Deposition and Annotation System (Young *et al*., 2017 ▸) now allows depositors to identify the location (doi) of their related experimental data set and its supporting metadata. Figure kindly provided by the RCSB Protein Data Bank and reproduced here with permission.

Most recently, the IUCr global Diffraction Data Deposition Working Group (DDDWG) has, over six years, examined the issues and prospects for linking raw diffraction data sets to publications in the modern era. Considerable progress has been made. The report for 2011–2014 can be found at http://bit.ly/2xU7nBz. A series of papers in *Acta Crystallographica Section D* (Terwilliger, 2014 ▸; Guss & McMahon, 2014 ▸; Kroon-Batenburg & Helliwell, 2014 ▸; Meyer *et al.*, 2014 ▸; Terwilliger & Bricogne, 2014 ▸) provides an overview of the reasons for archiving raw diffraction data, the practicalities and the potential benefits. It is also worth pointing out that the efforts of the DDDWG have stimulated the major development that the Protein Data Bank now dedicates a portion of their deposition-procedure web forms to details of where the raw diffraction data can be found (Fig. 1 ▸), and IUCr Journals (*IUCrJ*, *Journal of Applied Crystallography*, *Acta Crystallo­graphica Section D* and *Acta Crystallographica Section F*) have started linking their publications to primary crystallo­graphic data sets deposited in repositories. This provides the complete ‘version of record’, allowing succeeding researchers to review the entire scientific argument. Indeed, a strong case could be made for making the raw data available for inspection when the article is submitted for publication.

The significance of exposing all of the underlying data for review cannot be overstated. Since its inception, the IUCr Journals article-submission system has allowed the upload of coordinates and the processed data in the form of structure factors. The provision of such data to reviewers has been obligatory for some journals in chemical crystallography for a long time (since the 1990s) and has been a possibility in biological crystallography. Obviously, the advantage of this is that the editor and referees can consider a submitted article and the accompanying structure factors and atomic coordinates together and thus agree with the authors on an accepted ‘version of record’ of the three of these. One of us (JRH) recalls his early enthusiasm for providing experimental data for careful scrutiny because, while submitting his DPhil thesis to his examiners in 1977, he included the diffraction data on microfiche within his thesis! The widespread availability of deposited raw data sets can only help with the extension of reviewer scrutiny to as close as possible to the actual experiment.

## 4. Science case studies   

### 4.1. A chemical crystallography case study   

**Figure 2:**
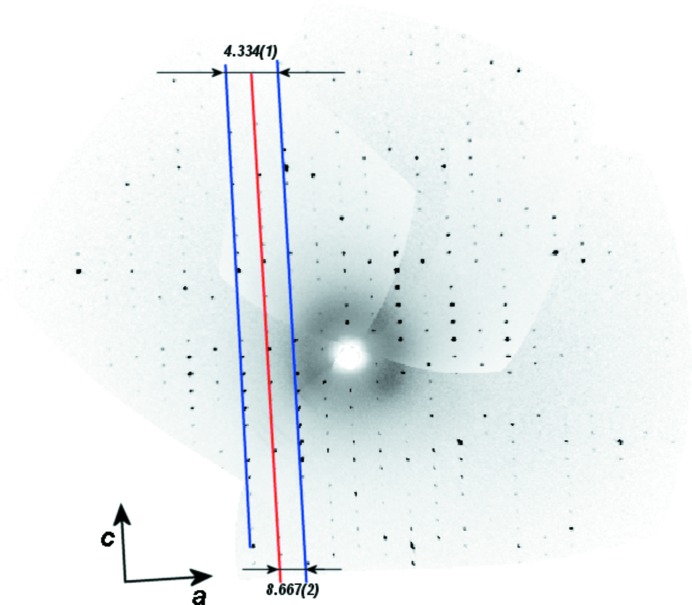
The revised crystal structure of *trans*-resveratrol involved a doubling of one of the unit-cell dimensions. There were weaker interleaving layer lines that were missed in the original analysis and the correct unit cell was established in the re-analysis. For details, see the text. Reproduced with the permission of the authors (Zarychta *et al.*, 2016 ▸) and the journal *Bioorganic and Medicinal Chemistry Letters*.

Zarychta *et al.* (2016 ▸) noticed that the crystal structure of *trans*-resveratrol reported by Caruso *et al.* (2004 ▸) included a dynamically disordered hydrogen-bonding network, which was shown by Zarychta *et al.* (2016 ▸) to instead be the superposition of two crystallographically independent molecules of *trans*-resveratrol. This latter arrangement possessed a well defined hydrogen-bonding network in a unit cell of double the previously reported volume (Fig. 2 ▸). This redetermination of the *trans*-resveratrol structure involved repeating all of the steps of the original study from purchase of the raw material to refinement and analysis of the structure, as the raw diffraction images were not available or more likely not even preserved. Zarychta *et al.* (2016 ▸) stated:

Initial crystallization experiments from ethanol–water solution confirmed the previously reported result, that is, thin plates with the smallest dimension of *ca.* 10 µm were obtained, however, the unit cell volume was observed to be double that previously reported.

It is unlikely that this is an isolated example of reinterpretations that could proceed swiftly and cheaply if the raw diffraction data were available.

### 4.2. A powder diffraction case study   

**Figure 3:**
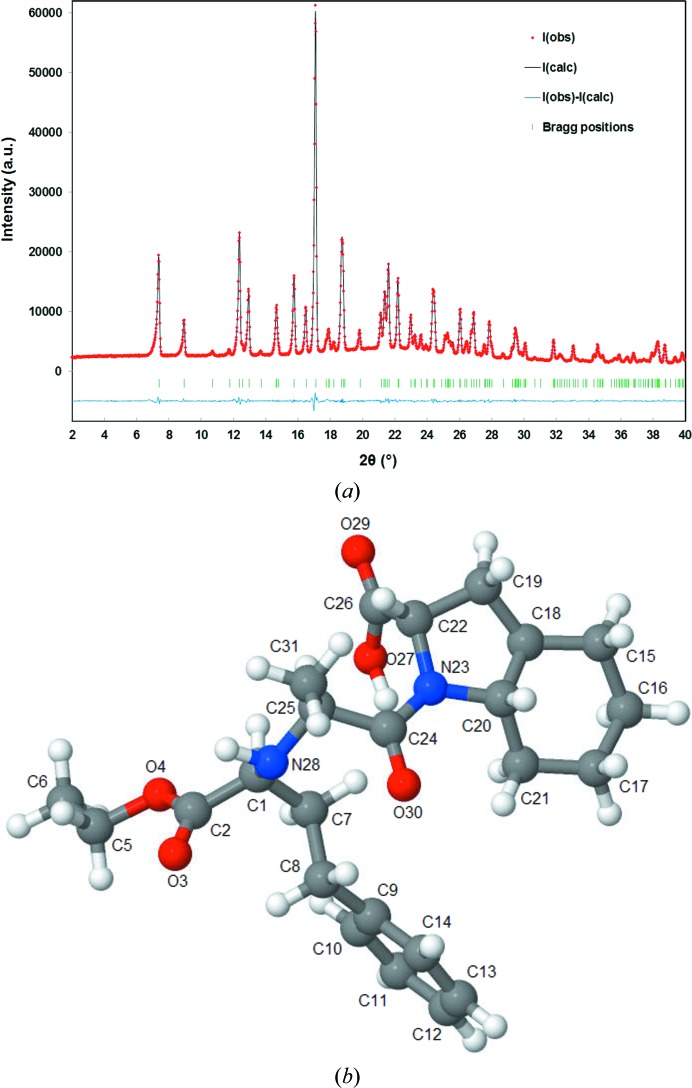
An example from the ICDD: (*a*) the high-quality powder diffraction data of the crystal structure of trandolapril (University College London) held in the PDF; (*b*) the crystal structure of trandolapril. Reproduced with the permission of Reid *et al.* (2016 ▸) and the journal *Powder Diffraction*.

Reid *et al.* (2016 ▸) undertook the crystal structure determination of trandolapril, C_24_H_34_N_2_O_5_, and showed the utility of raw data deposition in the powder diffraction file. The powder diffraction data for the crystal structure of trandolapril (from University College London) were of high quality (Fig. 3 ▸). Commenting on the benefits of retaining raw powder diffraction data in the PDF, an activity ongoing for many years, Reid *et al.* (2016 ▸) stated

This work illustrates one of the advantages of including raw data in the PDF, the potential for collaborative work within the powder diffraction community to solve new structures. Raw powder diffraction data also provide significantly improved illustration of materials with anisotropic broadening features or poor crystallinity such as clays, polymers and amorphous materials.

The International Centre for Diffraction Data (Reid *et al.*, 2016 ▸) also commented on the collection and then publication of raw powder diffraction data as follows:

While the International Centre for Diffraction Data (ICDD) has collected raw powder diffraction data for many years, submitted both by Grant-in-Aid recipients and private contributors, in the 2008 release of Powder Diffraction File PDF-4 products the ICDD began publishing raw data as part of both new and legacy PDF entries.

### 4.3. A macromolecular crystallography case study of collaboration and of critique   

**Figure 4:**
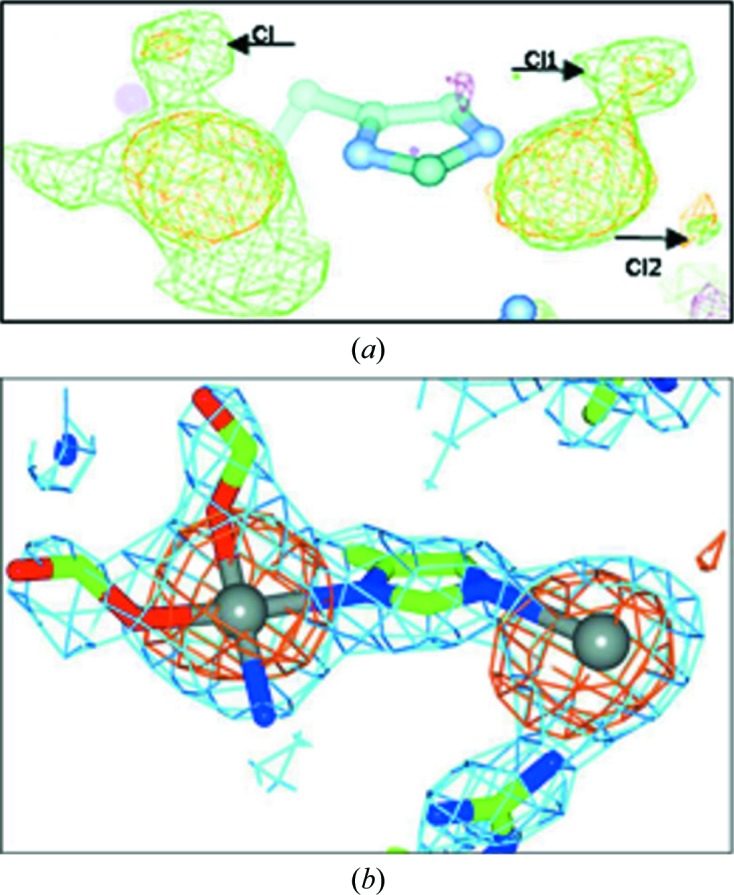
An example of the benefits of raw diffraction data sharing in macromolecular crystallography: avoiding the conversion of carboplatin to cisplatin at a high concentration of sodium chloride. (*a*) The partially converted carboplatin (Tanley, Diederichs *et al.*, 2013 ▸) and (*b*) alternative crystallization conditions led to the best study yet of carboplatin binding to histidine (Tanley *et al.*, 2014 ▸). Reproduced with the permission of the IUCr.

Kroon-Batenburg *et al.* (2017 ▸) describe their experiences in the archiving and sharing of raw diffraction images in a collaboration between Manchester and Utrecht Universities studying the binding of the important anticancer agents cisplatin and carboplatin to histidine in a protein. Through their raw data sharing (Tanley, Schreurs *et al.*, 2013 ▸), further analyses of raw diffraction images using *XDS* (Kabsch, 1988 ▸) were made by Dr Kay Diederichs, and a detailed crystallo­graphic assessment followed of the conversion of carboplatin to cisplatin under a high chloride salt concentration. This led to a new study, involving the crystallization of hen egg-white lysozyme with carboplatin under non-NaCl conditions, which was undertaken and published with Dr Diederichs (Tanley, Diederichs *et al.*, 2013 ▸; see Fig. 4 ▸).

The download of another raw data set from this work was involved in a sequence of structure revisions arising from the critique of Shabalin *et al.* (2015 ▸) of the whole field of the binding of cisplatin to various proteins. The subsequent revised and re-revised structures arising from this critique (Tanley *et al.*, 2016 ▸) provide a good example of the paradigm of ‘continuous improvement of macromolecular structure models’ (Terwilliger, 2012 ▸). For further comment on this example, see Kroon-Batenburg *et al.* (2017 ▸).

### 4.4. An example from charge-density analyses: the thermal diffuse scattering correction   

The diffuse scattering that peaks at the Bragg positions is that arising from phonons, and the number of unit cells participating in the phonon wave is necessarily limited and results in a broad peak that is seen even with home-laboratory sources; for a recent example and its successful correction, see Niepötter *et al.* (2015 ▸). Intriguingly, the softness of any particular sample will determine the number of unit cells involved in the phonon wave, *i.e.* soft crystals will engage fewer unit cells and thereby result in broader thermal diffuse scattering (TDS) peaks. A quantitative single parameter of the softness of a crystal and the likely behaviour of the phonon, and width of the TDS peak under a Bragg profile, is the speed of sound in a crystal, which can be measured, for example, by laser-generated ultrasound (see, for example, Edwards *et al.*, 1990 ▸). As pointed out by Niepötter *et al.* (2015 ▸), the use of liquid-helium temperature for X-ray diffraction data collection is also a method for reducing the TDS intensities under the Bragg peaks. Highly collimated synchrotron radiation also ameliorates the problem, as the Bragg reflection profile for a good-quality crystal is determined by the sample itself, rather than by a relatively uncollimated home-laboratory X-ray beam. The combination of liquid helium and highly collimated synchrotron radiation would provide the experimental methods of choice for the most accurate charge-density research using X-ray diffraction data as free as possible from TDS. The detailed and highly careful multiple avenues of investigations of Niepötter *et al.* (2015 ▸) illustrate that the methods of diffraction-image data processing are still maturing and again commend the value of preserving raw diffraction data for reuse.

### 4.5. A further example from charge-density research   

In an electron-density study of the linear iron(I) complex [K(crypt-222)]{Fe[C(SiMe_3_)_3_]_2_} (Thomsen, 2017 ▸), the structure of the selected compound published by Zadrozny *et al.* (2013 ▸) in a high-profile journal contained only one iron(I) complex in the asymmetric unit. The ensuing re-investigation study involved complete resynthesis of the compound, its crystallization and X-ray diffraction re-measurement and analysis featuring two geometrically distinct iron(I) complexes. The ellipsoids of the C(SiMe_3_)_3_ ligands from Zadrozny *et al.* (2013 ▸) were elongated in the directions complying with a rotation of one of the C(SiMe_3_)_3_ ligands with respect to the other around the C—Fe—C line. These elongated ellipsoids obviously resulted from the fact that the molecular geometry in their model was an average of two correct geometries with Si—C—C—Si torsion angles ∼8° above and below the average angle of ∼22°. The probability ellipsoids of the C(SiMe_3_)_3_ ligands from the structure determined by Thomsen (2017 ▸) are smaller and more isotropic, although the temperature during data collection was 100 K in both studies. Thomsen (2017 ▸) concluded that the previously published structure was most likely to have been obtained by overlooking the superstructure reflections. The availability of the raw diffraction data would have made a reanalysis directly feasible, and this could have been readily undertaken by the referees of the original submitted article.

### 4.6. A corrigendum case in protein crystallography   

An interesting survey of crystallographic retractions is given by Retraction Watch. Their news item ‘*Structural biology corrections highlight best of the scientific process*’ (http://retractionwatch.com/category/by-subject/basic-life-sciences-retractions/crystallography-retractions) discusses the case of the correction of an earlier publication by the Blumberg group in *Nature* (Huang *et al.*, 2015 ▸) prompted by criticism by E. Sundberg. The original crystal structure was determined as a heterodimer of the human hCEACAM1 IgV domain and hTIM-3 IgV domain (PDB entry 4qyc) by molecular replacement at a resolution of 3.4 Å. Sundberg and Almo were aware of the strong tendency of CEACAM1 to form homodimers and observed that the refinement statistics of PDB entry 4qyc were exceptionally poor. They retrieved the structure-factor data from the PDB and found that the statistics were much improved by fitting a homodimer in the crystal lattice. The Blumberg group believe that the low resolution and similarity between the folds of hCEACAM1 and hTIM-3 led to a failure by the authors to realise that they were using the wrong model. Subsequent further analysis showed that hCEACAM1 binds hTIM-3, but that in the crystallization step hTIM-3 was apparently the subject of proteolysis and the strong self-association interaction of hCEACAM1 resulted in a crystal of the homodimer. The corrected structure has been deposited in the PDB as entry 5dzl (Huang *et al.*, 2016 ▸) and a search for 4qyc in the PDB is automatically redirected to PDB entry 5dzl. This is a scholarly example of how the scientific process should work: critique leads to a more in-depth study.

### 4.7. Critical analysis of ligand density   

Very recently, a message was posted on the CCP4 bulletin board (Tanner, 2017 ▸) about the correction of a misplaced ligand-binding site by complete resynthesis, crystallization and structure determination of the proline-biosynthetic enzyme PYCR1 (PDB entry 5uat; Christensen *et al.*, 2017 ▸). The original structure PYCR1 (PDB entry 2gr9; Meng *et al.*, 2006 ▸) was solved at low resolution (3.1 Å); it is a pentameric dimer complex and, contrary to what was expected, the NADH ligand did not bind at the canonical NAD(P)H-binding site at the C-termini of the strand of the Rossman fold, but 25 Å away from that in the dimer interface. Careful analysis by the Tanner group showed that in the 2gr9 structure strong negative difference densities were present at all five NADH-binding locations and the *B* factors for the ligand were exceptionally high. The Tanner group obtained crystals that diffracted to 1.9 Å resolution, and two NADPH ligands could very clearly be built into the expected canonical binding site as supported by additional biophysical studies. Thus, the record on the ligand binding pyrroline-5-carboxylate reductase was corrected.

### 4.8. Reinterpretation of data in macromolecular crystallography   

The crystal structure of lipoxygenase 15S-LOX1 showed unexpected disorder in a long α-helix and many residues could not be modelled into the density (Gillmor *et al.*, 1997 ▸). Choi *et al.* (2008 ▸) knew of a study by Oldham *et al.* (2005 ▸) who had found significant disagreement between the structures of 8R-LOX and 15S-LOX. Close inspection by Choi and coworkers showed that two helices related by a crystallographic twofold axis actually collided. The structure-factor data were downloaded from the PDB; these were merged in *R*32 symmetry. The authors reinterpreted the structure as perfectly twinned in space group *R*3, thus relieving the symmetry constraint between two neighbouring molecules. This resulted in significantly differing conformations of the α-helices and resulted in well defined electron densities. Statistical analysis of unmerged structure-factor data would have been possible in the present-day regime of unmerged data deposition in the PDB, although in the case of perfect twinning in *R*3 it would be hard to discern from *R*32 symmetry.

A further, very recent and rather extreme, case of incorrect publication of protein structures, and admission into the PDB, is described by Weiss *et al.* (2016 ▸), who describe their critical complete re-examination of the crystal structure of supposedly human survival motor neuron (SMN) protein. Seng *et al.* (2015 ▸) reported the structure of full-length SMN protein (PDB entry 4nl6) obtained by molecular replacement using their structure of SMNΔ7 (PDB entry 4nl7), which was in turn based on their SMN1-4 structure, for which no PDB coordinates or structure factors were deposited. Amongst other problems, hydrophobic side chains point outwards instead of inwards to the hydrophobic core, and backbone–backbone interactions in helices and β-sheets are distorted or absent. The molecular-replacement calculations could not be repeated because the SMN1-4 data had not been published. Meticulous detective work led Weiss and coworkers to hypothesize that the crystal was actually of the bacterial protein Hfq that could be co-expressed in *Escherichia coli*, the crystal structure of which has similar unit-cell parameters (once the *C*2 symmetry constraint of PDB entry 4nl7 is released). The Hfq model fitted with a 10% better *R*_free_. In a similar way, Weiss and coworkers were able to prove that the full-length SMN protein crystal structure was in fact that of the Gab protein from *E. coli*.

A similar problem with the crystallization of a contaminant protein was described by Hatti *et al.* (2017 ▸). Mutants of a survival protein from *Salmonella typhinurium*, StSurE, were expressed and crystallized. The unit-cell parameters were different from those of StSurE, but this structure or domains thereof were used in molecular replacement. The resulting structure, obtained from 3.0 Å resolution data, however, was not satisfactory: many parts of the backbone fitted into the electron density, but the *R* factors did not improve beyond 35%. Therefore, the authors resorted to the *MarathonMR* molecular-replacement software, in which representative structural domains from the SCOPe database were used. The highest score was obtained with a dehydroquinate synthase-like fold. After reprocessing the data to higher resolution and extensive model building, the structure was identified to be *Entero*GlyDH. The paper shows how hard it is to solve a structure for which the sequence is not known, and that automatic structure solution with molecular replacement could have come up with the wrong structure.

### 4.9. An example from physical crystallography: a new theory for X-ray diffraction   

Fewster (2014 ▸, 2016 ▸) has proposed a new theory for X-ray diffraction; this (we quote)

…new theory of X-ray scattering has particular relevance to powder diffraction. The underlying concept of this theory is that the scattering from a crystal or crystallite is distributed throughout space: this leads to the effect that enhanced scatter can be observed at the ‘Bragg position’ even if the ‘Bragg condition’ is not satisfied. The scatter from a single crystal or crystallite, in any fixed orientation, has the fascinating property of contributing simultaneously to many ‘Bragg positions’. It also explains why diffraction peaks are obtained from samples with very few crystallites, which cannot be explained with the conventional theory… This theory, when applied to the scattering from powders, will evaluate the full scattering profile, including peak widths and the ‘background’… The intensity is heavily dispersed outside the Bragg condition in both … and, therefore, so is the scattering power.

The verification of this new theory by a wide range of samples suggests that routine archiving of raw diffraction data ideally as a diffraction image rather than a one-dimensional profile would be beneficial.

## 5. Discussion   

These science case examples show the growing interest in reusing derived data (structure factors) and in some cases show that scientific validation would require re-evaluation of raw diffraction data. They demonstrate the potential benefit of preserving raw diffraction data and making them accessible. We envisage that in the future, with the availability of raw diffraction data, re-evaluations will probably have even larger impact and improve the soundness of crystallographic science.

The availability of raw diffraction data is a revolution for the crystallographic community and will have its impacts in the future for our further education, *i.e.* continual professional development as crystallographers. Firstly, researchers need to learn the new protocols associated with archived, open, raw diffraction data, as well as the processed diffraction data and derived coordinates with which they are quite familiar. Within the ‘archived, open, raw diffraction data’ approach the funding agencies are looking at an Open Science protocol for improvements to the speed of discovery, especially with respect to societal challenges, including ‘the sharing of data right from the start of a funded research project’.

Furthermore, as an educational benefit of access to raw diffraction images, we can reflect that a community that publishes detailed ontologies of all aspects of their data workflow (*i.e.* in our case the Crystallographic Information Framework ‘CIF’ dictionaries) makes the concepts that are important in collecting, categorizing and analysing the data quite transparent. These provide very valuable material for scientists to deepen their understanding of all aspects of data analysis and, as a result, become more critical and careful in undertaking their analyses. An example in macromolecular crystallography that emphasizes this is from Grabowski *et al.* (2016 ▸), who will analyse data sets that are non-optimal, such as from the inappropriate use of too large a rotation range in monochromatic data collection or synchrotron beamlines with a below-average results output.

The area of data analysis in macromolecular crystallo­graphy, for which we have highlighted several case studies above, has been attracting wide attention for a long time. An independent initiative that has now been running for many years is the *PDB_REDO* project (Joosten *et al.*, 2014 ▸). This provides a rerefined (*i.e.* new coordinates) set for each and every PDB deposition. It also offers a useful server to assist the depositor to look at the *PDB_REDO* version of their current cycle of model refinement before deposition. Whilst the PDB validation report is a vital part of modern crystal structure article peer review, unfortunately in many situations the PDB summary validation reports are insufficient to pinpoint the validity of the claims made by an article. There is then a concern over the occurrence of ‘bad apples’ admitted into and released by the structural repositories (Minor *et al.*, 2016 ▸). Weichenberger *et al.* (2013 ▸) and Pozharski *et al.* (2013 ▸) have critically examined the whole area of visualizing ligand molecules in ‘twilight electron density’. A number of case studies of rebuilding ligands in PDB entries is provided by Smart & Bricogne (2015 ▸), based on poor electron densities and issues with ligand geometries. In addition, examples are shown of the consequence of incomplete data for the ligand density, be it owing to an incorrect data-collection strategy or suboptimal data processing. Their analysis led to several PDB redepositions. The authors advocate the benefits of reprocessing raw diffraction data, *via* its archiving, as ‘many mistakes can be made at the data integration and other stages during the processing’. These highlight more than just a few examples but expose the need for greater vigilance by journal editors in scrutinizing the articles that they accept (Rupp *et al.*, 2016 ▸). The insistence by some journals that the PDB validation report be provided by authors on submission is a major step in the right direction. In line with these stricter validation procedures, referees could be required to have sufficient skills to assess the model refinement of the processed data, and if needed to require the authors to reprocess the raw data according to their (the referee’s) prescription or themselves if they wish (Helliwell, 2017 ▸).

## 6. Summary   

This article has provided an overview of the preservation of raw diffraction data. It has documented through a range of science case studies, as examples drawn across the various IUCr scientific Commissions, the potential for use of digital archives to generate a revolution in our activities. High-volume robust storage archives constitute a wonderful resource that has now become available to us.

The points of philosophy and practice underlying the motivations for pursuing opportunities for raw diffraction preservation and reuse have shown an interesting variation in the last six years since the DDDWG was formed in 2011 by the IUCr Executive Committee.

There has, of course, been the initial practical difficulty of embracing routine deposition of primary data, because of the size of the raw data sets that crystallographers work with, their typical number in any year’s worth of research activity, and the fact that the crystallography community is quite large (∼15 000 people are registered in the IUCr World Directory of Crystallographers; https://www.iucr.org/people/wdc).

With the exception of the ICDD, as described above in §[Sec sec4.2]4.2, the existing crystallographic data archives were unwilling to take up the scale of the challenge because of cost and also because there was a lack of community consensus for it. However, happily, the crystallographic databases took part in or advised the DDDWG, a not unreasonable stance by established database organizations. Journals were reluctant to take up the ‘opportunity’ of accepting raw diffraction data transferred to them with an article even though they could see the philosophically compelling argument of linking raw data to a publication (Strickland *et al.*, 2008 ▸). They were concerned with storage capacity and the network-bandwidth overload associated with such an extension of their function. An exception is where smaller data-set files exist, such as small-angle X-ray scattering (SAXS), where the data are attached to the article; for an example, see Rhys *et al.* (2011 ▸). As a piece of historical context, it is worth noting that in the earlier days of IUCr Journals tables of derived structure-factor data were typically published as photographs of computer-generated tables in every crystal structure paper. At the time there was no computer storage for even the processed data!

The funding agencies, whilst developing research data-management policies and impositions on their funded research grant holders, did not wish to be responsible for paying for data archives. The view of the UK Universities was to take up the responsibility for data archiving. Here, the University of Manchester, as a major research player, was one of the pioneers. A major overarching initiative within a stunning vision of openness for the good of improved and more speedy research for the benefit of society (Moedas, 2016 ▸) is the Zenodo archive described above.

Overall, we emphasize that all data *including the raw data* should be made available to readers of the scientific literature to enable them to check all of the decisions made by authors in a given research study. Not least, ***the science is in the data***!

## Figures and Tables

**Table 1:** Summary of case studies in this article The categories ‘sharing’, ‘preservation’ and ‘no data’ correspond to the benefits or hindrances to scientific progress described in the final sentence of §[Sec sec1]1.

Section	Structure/topic	Field	Category
§[Sec sec4.1]4.1	*trans*-Resveratrol	Chemical crystallography	No data
§[Sec sec4.2]4.2	Trandolapril	Powder diffraction	Sharing
§[Sec sec4.3]4.3	Cisplatin/carboplatin	Macromolecular	Sharing
§[Sec sec4.4]4.4	TDS correction	Charge density	Preservation
§[Sec sec4.5]4.5	Linear iron complexes	Charge density	No data
§[Sec sec4.6]4.6	hCEACAM1/hTIM-3	Macromolecular	Sharing
§[Sec sec4.7]4.7	Proline-biosynthetic enzyme	Macromolecular	Sharing
§[Sec sec4.8]4.8	Lipoxygenase	Macromolecular	Sharing
§[Sec sec4.8]4.8	Survival motor neuron protein	Macromolecular	Sharing
§[Sec sec4.8]4.8	*S. typhimurium* StSurE	Macromolecular	Preservation
§[Sec sec4.9]4.9	Non-Bragg scattering	Physical crystallography	Sharing
